# Migratory herbivorous waterfowl track multiple resource waves during spring migration

**DOI:** 10.1098/rspb.2024.1448

**Published:** 2024-09-11

**Authors:** Fei Xu, Wei Wu, Jie Wei, Qinchuan Xin, Ben Wielstra, Frank A. La Sorte, Zhijun Ma, Guangchun Lei, Jialin Lei, Wenzhao Wu, Yongchuan Yang, Peng Gong, Bing Xu, Yali Si

**Affiliations:** ^1^ Key Laboratory of the Three Gorges Reservoir Region’s Eco-Environment, Ministry of Education, Chongqing University, Chongqing 400045, People’s Republic of China; ^2^ Department of Earth System Science, Ministry of Education Field Research Station for East Asian Migratory Birds, Tsinghua University, Beijing 100086, People’s Republic of China; ^3^ Mining College, Guizhou University, Guiyang, Guizhou 550025, People’s Republic of China; ^4^ School of Geography and Planning, Sun Yat-sen University, Guangzhou 510275, People’s Republic of China; ^5^ Institute of Biology, Leiden University, Leiden, The Netherlands; ^6^ Naturalis Biodiversity Center, Leiden, The Netherlands; ^7^ Department of Ecology and Evolutionary Biology, Yale University, New Haven, CT 06511, USA; ^8^ Center for Biodiversity and Global Change, Yale University, New Haven, CT 06511, USA; ^9^ Ministry of Education Key Laboratory for Biodiversity Science and Ecological Engineering, Coastal Ecosystems Research Station of the Yangtze River Estuary, and Shanghai Institute of Eco-Chongming (SIEC), Fudan University, Shanghai 200433, People’s Republic of China; ^10^ School of Ecology and Nature Conservation, Beijing Forestry University, Beijing 100083, People’s Republic of China; ^11^ Department of Geography, Department of Earth Sciences, Institute for Climate and Carbon Neutrality, The University of Hong Kong, Hong Kong; ^12^ Institute of Environmental Sciences, Leiden University, Leiden 2333 CC, The Netherlands

**Keywords:** resource tracking, seed wave, onset of vegetation green-up, agricultural land, migration phenology, seasonal bird migration

## Abstract

East Asian herbivorous waterfowl intensively use farmland in spring, next to their natural habitat. Accordingly, they might have expanded their migration strategy from merely tracking the green wave of newly emerging vegetation to also incorporating the availability of post-harvest agricultural seeds (here dubbed the seed wave). However, if and how waterfowl use multiple food resources to time their seasonal migration is still unknown. We test this migration strategy using 167 spring migration tracks of five East Asian herbivorous waterfowl species and mixed-effect resource selection function models. We found that all study species arrived at their core stopover sites in the Northeast China Plain after agricultural seeds became available, extended their stay after spring vegetation emerged and arrived at their breeding sites around the emergence of vegetation. At the core stopover sites, all study species used snowmelt as a cue to track seed availability, although smaller-bodied species tended to arrive later. At the breeding sites, swans tracked the onset of vegetation emergence and geese tracked the mid- or end phases of snowmelt. Our findings suggest that waterfowl track multiple resource waves to fine-tune their migration, highlighting new opportunities for conservation.

## 1. Introduction

Migratory species move between their breeding and non-breeding grounds and exploit seasonal food resources across their annual cycle [1]. Foraging opportunities occur in a series of pulses, at different times and places, which generate ‘resource waves’ across the landscape [1–5]. The green wave hypothesis posits that the migration progress of herbivores is driven by the seasonal growth of new vegetation at stopover sites along the migration route [6,7]. Studies have verified this hypothesis for a variety of herbivorous animal groups, such as geese [4,6,8–10] and ungulates [11–13]. Evidence has not only accumulated from individual-level information compiled using tracking technology [6] but also from population-level information compiled by large-scale community science initiatives [14]. During spring migration, European goose populations mainly arrive at their stopover sites around the peak in nutrient biomass (plants with the highest amount of nitrogen per unit area) but can reach their breeding grounds earlier (around the emergence of spring vegetation), thus overtaking the green wave, which allows their offspring to benefit from the peak of food resource that occurs later [4]. While herbivorous species have been documented to track the green wave in northern Europe [6,10,15] and North America [14], this does not apply worldwide, especially within East Asia [15].

Due to extensive changes in global land cover, herbivorous waterfowl have expanded from using only natural ecosystems to exploiting food resources in agricultural landscapes [16,17]. For example, during the spring-staging period, herbivorous waterfowl have been observed to consume newly sown cereals in Europe [18], post-harvest corn remaining in cornfields, and spilled grain in rice paddies in North America [19–22] and in Asia [23–28]. This way, they benefit from the post-harvest management of agricultural lands [29,30]. East Asian waterfowl populations intensively use the Northeast China Plain during migration [23], where large areas of natural wetland have been converted into farmland since the 1950s [31–34]. As a consequence, East Asian waterfowl may take the availability of both food resources into account during spring migration. The timing of resource peaks in natural habitats and on farmlands likely differ, which would make it profitable for waterfowl species to exploit both resources. However, this possibility of tracking multiple resource waves during migration has not yet been investigated.

Migratory herbivorous waterfowl use the natural habitat (e.g. wetlands, grasslands and shrublands) and farmland habitat differently. Since many waterbodies and grasslands in East Asia are semi-natural, we use the term ‘natural habitat’ hereafter to represent areas where birds feed on spring sprouts of vegetation (representing the green wave). Within farmlands, East Asian waterfowl primarily consume seeds, rather than crop vegetation, e.g. spilled grains and leftover maize that remained after the harvest in the previous autumn and so have been carried over into spring [23]. These seeds, in general, offer higher food quality in terms of energy and protein, and lower fibre compared with natural vegetation [16,29].

Spilled seeds are available after the harvest in autumn but not in winter when the ground is frozen and covered in snow. In spring, the remaining seeds become available again as snow melts, forming a second peak, that we dub the seed wave (electronic supplementary material, figure S1). At the core stopover area of the Northeast China Plain, the daily minimum temperature in winter can drop below −25℃ [35,36]. It often starts snowing in November and ends in late March [35,37]. This makes the post-harvest seeds inaccessible in winter, but seeds can be exploited by waterfowl after the ground thaws in late March [37]. The seed wave then ends at the onset of sowing in early May [37], due to pesticides being mixed in with newly planted seeds (electronic supplementary material, figure S2*c*). The timing of seed availability starts before, and partially overlaps with, the emergence of vegetation [15]. How the herbivorous waterfowl time their migration based on these two resource waves is still unclear.

**Figure 1.**
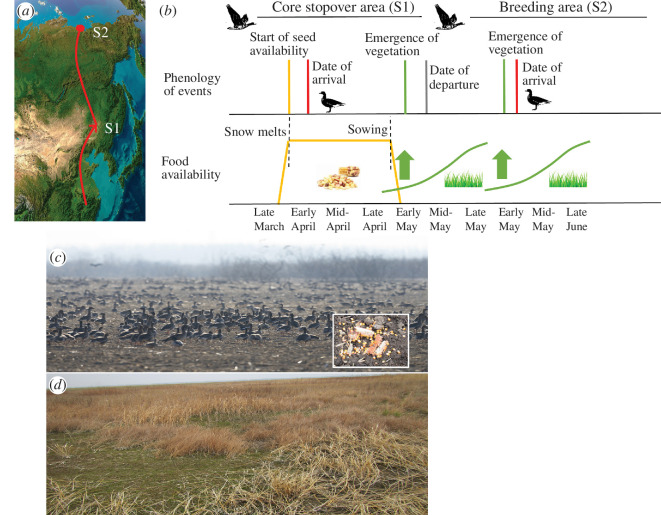
Hypothesized responses of tracking multiple resource waves by East Asian migratory herbivorous waterfowl. (***a***) Core stopover area (S1) and breeding area (S2) along the spring migration route. (*b*) During spring migration, we hypothesize that birds arrive on farmlands at the core stopover area after the start of seed availability (seed wave; orange line), extend their stay after the emergence of spring vegetation in natural habitats (green wave; green lines) and reach the breeding area after the emergence of vegetation (green wave; green lines). (*c*) Photo of greater white-fronted geese (*A. albifrons*) and tundra bean geese (*A. serrirostris*) foraging in farmlands (taken on 19 March 2021) on Yuquan Island, Hunchun, Yanbian Korean Nationality Autonomous Prefecture, Jilin, People’s Republic of China (photo credit: Haixiang Zhou). Inset photo shows leftover maize (taken 2 May 2016) on farmlands in Fuyu village, Nehe, Qiqihar, People’s Republic of China (photo credit: Jie Wei). (*d*) Photo of new spring vegetation (taken on 1 May 2016) embedded in old vegetation in Baishan town, Longjiang, Qiqihar, People’s Republic of China (photo credit: Jie Wei).

We aim to investigate if and how herbivorous waterfowl track multiple resource waves during spring migration. We analyse 167 spring migration tracks from 2015 to 2021, derived from satellite tracking of 99 individuals for five herbivorous waterfowl species (swan goose *Anser cygnoides*, tundra bean goose *Anser serrirostris*, greater white-fronted goose *Anser albifrons*, lesser white-fronted goose *Anser erythropus* and tundra swan *Cygnus columbianus*) in East Asia, using a mixed-effect resource selection function (RSF) model. We hypothesize that migratory herbivorous waterfowl time their arrival at stopover and breeding areas based on the availability of multiple food resources in natural habitat and farmland (figure 1). We predict that: (i) all study species time their migration to use seeds in farmland upon arrival at the core stopover area, later shift to natural habitat after the vegetation emerges and arrive at the breeding area around the time of vegetation emergence, (ii) waterfowl tend to track the start of seed availability (i.e. the seed wave) at their core stopover area and then follow the newly emerged plants (i.e. the green wave) as a cue to arrive at their breeding area (figure 1). Understanding how herbivorous waterfowl use the spatio-temporal availability of multiple food resources during migration is fundamental to predict species’ responses to land-use change and climate change. The wider implications of our findings are new opportunities for the effective management of multiple types of habitats to conserve migratory waterfowl.

## 2. Material and methods

### (a) Satellite tracking of herbivorous waterfowl

Lesser white-fronted geese (*A. erythropus*), greater white-fronted geese (*A. albifrons*), tundra bean geese (*A. serrirostris*) and tundra swans (*C. columbianus*) were captured with leg nooses and flat nets at their East Dongting Lake (29°N, 113°E) and Poyang Lake (29°N, 116°E) wintering sites in the Yangtze River Floodplain, People’s Republic of China, from 1 January to 2 March and from 16 November to 31 December during the 2014 to 2018 wintering seasons. Swan geese (*A. cygnoides*) were captured with flat nets at the breeding site Hulun Lake, Hulun Buir, People’s Republic of China (48.3°N, 117.4°E), from 8 July to 16 July during the 2017 moulting season. Birds were placed individually into bags and immediately transported to the closest handling station.

All birds were equipped with GPS–GSM (global positioning system–global system for mobile communications), solar-powered neck-band loggers (electronic supplementary material, table S1). The weights of the satellite transmitters range from 22 to 72 g, corresponding to 0.3–2.3% of an individual bird’s body weight (electronic supplementary material, table S1 and table S2), which falls under the rule of thumb that loggers should weigh less than 3% of body weight [38]. Birds were released at the capture site after an average of 6 h [23]. GPS locations were recorded every 2 h. Information used in the analysis included bird ID, tracking year, longitude, latitude and time of the GPS location. Detailed methods for bird capture and deployment of the satellite transmitters can be found elsewhere [23,39]. Previous studies reported various effects of tagging on the behaviour of waterfowl. Short-term effects could last a few days for geese [40] and 6 weeks for swans [41]. Geese were tagged at least 5 days and swans at least 6 weeks before the onset of spring migration. We thus expect a minimal short-term effect of tagging on migration behaviour. Indeed, we found no obvious change of migration distances between the first year that birds were tagged with the following years (*p* > 0.05; electronic supplementary material, figure S3 and figure S4). Long-term effects on migration timing and reproduction have also been reported [42–44]. Slight delays in migration onset and egg laying were found for tagged goose individuals compared with untagged ones [42]. Such effects are expected to equally influence individuals and thus it would not change the results of this study.

### (b) Analysing bird migration patterns

We quantified migration parameters (electronic supplementary material, table S3 and figure S5) in three steps. First, we subset all GPS locations between 1 January and 31 July, thereby including months of the whole spring migration period (February–June) and the months in which all five herbivorous waterfowl species arrived at breeding sites (June–July). The definition of the spring migration period is GPS locations recorded from the day that birds left their wintering sites to the day they reached their breeding sites [8]. Second, we labelled daytime and night-time locations, by identifying the sunrise and sunset time for each location based on algorithms provided by the National Oceanic & Atmospheric Administration (NOAA) (https://www.esrl.noaa.gov/) [45]. Daytime and night-time locations were defined as the locations recorded in the periods between 1 h before sunrise to 1 h after sunset, and 1 h after sunset to 1 h before sunrise [24].

Third, we identified stopover sites and the length of stay at each site through the space-time permutation model (electronic supplementary material, text) in SaTScan statistics (http://www.satscan.org) [23,46]. We defined the stopover sites as those sites where birds stayed for at least 2 days [47] and moved within a radius of less than 50 km [23,47–49]. The arrival/departure time of the wintering/stopover/breeding sites for each individual in each tracked year were defined as the first day it arrived/left a specific site in which it showed a continuous presence/absence [24]. The cumulative length of stay in the Northeast China Plain and Russian stopover sites was defined as the sum of all days spent at stopover sites by an individual in their respective region. Core stopover sites are defined as those located between 38.7 and 52°N latitude in the Northeast China Plain, where the cumulative length of stay exceeded half of the total migration period. Migration distance was defined as the cumulative distance travelled between the wintering and the breeding sites [23]. The total migration period was defined as the cumulative number of days travelled between the wintering and breeding sites [23].

We calculated mean dates of migration timing upon arrival at or departure from wintering sites, core stopover sites and breeding sites for all tracks of each species, i.e. (i) mean departure date from the wintering grounds, (ii) mean arrival date at core stopover sites, (iii) mean departure date from core stopover sites and (iv) mean arrival date at breeding sites. For each of the four types of migration timing, we compared the dates among five herbivorous waterfowl species in relation to their body size, which was obtained from EltonTraits 1.0 [50]. Differences in migration timing across species were assessed using one-way ANOVA followed by Tukey’s honestly significant difference (HSD) test for multiple comparisons.

### (c) Investigating resource utilization in migration

To examine habitat use along the spring migration route, we used two land cover products: the 30 m spatial resolution spring land cover product [51] from 2015 to capture the natural habitats and farmlands within People’s Republic of China and the European Space Agency Climate Change Initiative (ESA-CCI) 300 m spatial resolution land cover product from 2015 (http://maps.elie.ucl.ac.be/CCI/viewer/index.php) to capture the relatively homogeneous landscape within Russia. Two classification systems from the two products were aggregated into seven land cover types: grassland, farmland, water, wetland, forest, shrubland and ‘other’ (i.e. barren land, snow and ice, and built-up areas). Habitat use at each stopover site was estimated using the percentage of bird GPS locations on each land cover type [23]. Habitat use at each latitude was estimated using the percentage of bird GPS locations on each land cover type across all stopover sites located at the same latitude (rounded to 0 decimal degrees). The proportion of land cover types in use at each latitude was then calculated as the percentage of bird GPS locations on each land cover type, divided by the sum of the percentage of bird GPS locations across all land cover types at the corresponding latitude (electronic supplementary material, figure S6).

As waterfowl mostly forage during the day, we also calculated habitat use during daytime (i.e. the proportion of land cover in use during the day) across the whole spring migration period, to better interpret how waterfowl use the food resources across time. We investigated weekly habitat use during spring migration to see when species switch their habitat use from mainly farmlands to mainly natural habitats. We identified the habitat switch week, defined as the week before the first week when natural habitats become utilized more intensely than farmlands. To better investigate the multiple resource use across time within the core stopover sites in the Northeast China Plain, we divided the length of stay (electronic supplementary material, table S3) of herbivorous waterfowl into two periods: (i) from the arrival date at each stopover site in the Northeast China Plain to the local emergence of vegetation and (ii) from the local emergence of vegetation until they depart from this region. We then calculate the foraging distribution (i.e. the proportion of GPS locations during the day on farmlands and natural habitats) for all study species during these two different time windows (electronic supplementary material, figure S7). Finally, we tested the differences in habitat use in natural habitats and on farmlands using Welch’s two-sample *t*‐test for two situations: (i) before and after the habitat switch week during spring migration, and (ii) before and after the emergence of vegetation in the Northeast China Plain.

### (d) Calculating the timing of the seed wave and the green wave

The spring vegetation emergence time in natural habitats and the relative seed availability time on farmlands were calculated for each 250 m grid cell where birds were present. We used the second-order derivative [52,53] of the smoothed time series of the enhanced vegetation index (EVI), derived from the Moderate Resolution Imaging Spectroradiometer (MODIS) Terra 8 day 250 m spatial resolution surface reflectance product MOD09Q1, to extract the spring vegetation emergence time (details are provided in electronic supplementary material, text and figure S8). Only pure pixels [54] where the proportion of herbaceous area (i.e. wetlands, grasslands and shrublands) was larger than 0.5 within a 250 m grid cell were used (details are provided in electronic supplementary material, text and figure S9).

The relative seed availability time refers to the differing degree of snowmelt in spring. The higher the degree of snow melt, the higher the number of seeds (relative seed availability) that could be dug out from the ground by waterfowl (electronic supplementary material, figure S10). Following the method of Rickbeil *et al*. [55], we used the MODIS Terra Snow Cover daily 500 m spatial resolution product MOD10A1 to measure the differing degree of snowmelt, i.e. the early (25%), medium (50%), late (75%) phases and the end of snowmelt (100%) to represent four levels of relative seed availability (25, 50, 75 and 100%). We first created a time series to capture the daily percentage of snow cover (from day 1 to day 210) for each stopover and breeding site, for each track. We then applied three-piece segmented regression to extract two breakpoints for each pixel (electronic supplementary material, figure S11). Based on the fitted daily percentage of snow cover from the three-piece segmented regression, we derived the 25, 50, 75 and 100% relative seed availability date (electronic supplementary material, figure S11*a*) at core stopover sites. To facilitate interpreting the resource use, in addition to the vegetation availability date and the relative seed availability date, we also calculated the early, medium and late phases and the end of snowmelt (electronic supplementary material, figure S11*a*) at the breeding sites for each pixel where birds were present, for each individual bird, in each tracked year.

### (e) Determining the resource availability—bird arrival relationship

For each species, we used boxplots to show the resource and migration timing in different regions: (i) the emergence of vegetation and the timing of departure at wintering sites, (ii) relative seed availability (using 25, 50, 75 and 100% of snowmelt as surrogates), the emergence of vegetation, and the timing of arrival and departure at core stopover sites in the Northeast China Plain, (iii) snowmelt conditions (25, 50, 75 and 100%), the emergence of vegetation and the timing of arrival at breeding sites. We also tested the significance level of these differences using Welch’s two-sample *t*‐test.

We used a RSF framework [56,57] to investigate to what extent the start of resource availability can be matched with the arrival date at the stopover or breeding sites for migratory herbivorous waterfowl during spring migration. Using a logistic regression of presence/absence points, RSFs can be used to define the selection of resources on the day when waterfowl arrive at a specific site [24]. The presence points were defined as all GPS locations recorded on the day of arrival. For each site, we randomly drew absence dates from 1 January to 31 July (day 1 to day 211), excluding the day of arrival of this specific site used by the tracked individual in a tracked year. For each pixel in the core stopover or breeding sites, we calculated the number of days between each of the five indices (i.e. the spring vegetation emergence, the early (25%), mid (50%), late (75%) and full (100%) snowmelt) and the bird arrival date.

Migratory waterfowl might not track the exact day that a specific resource becomes available but may display a lag effect, where birds arrive before (positive lag value) or after (negative lag value) the resource becomes available [15]. In order to obtain the focal lag (i.e. the number of days before or after the resource available or snow melt dates), we tested the predictive ability of the models across a series of temporal lags, by adding or subtracting days to the resource becoming available or the snow melt dates, following Laforge *et al*. [57]. For example, if birds arrive at the stopover site 2 days after 75% snowmelt, the model with a lag of two (by subtracting two from the day to the 75% snowmelt date) would have the highest likelihood.

We executed RSFs in two regions for each species: the core stopover area of the Northeast China Plain and breeding sites at the mid-latitude (around 46°–49°N) or Arctic (around 70°−75°N), using vegetation availability and the date of 25, 50, 75 and 100% snowmelt as predictors. Snowmelt condition in the Northeast China Plain was used as a surrogate of seed availability. The response variable was the probability of bird arrival. The presence was set to 1 and absence to 0. For each of the five waterfowl species, within each region, we fit a mixed-effects conditional logistic regression model using a binomial distribution as the link function. The fixed effect for each model was a specific type of temporal lag, i.e. lag days to the spring vegetation emergence, the start of 25, 50, 75 or 100% snowmelt. For each individual tracked in each year, we obtained the identity of the track (track ID), named as individual (bird ID) and year (ID year). The track ID was included in the model as a random effect. We used the R package glmmTMB [58] to run the analyses.

We modelled the lag in days to vegetation availability or the snow melt conditions that fell between the 10th and 90th percentile of all locations within each region used by herbivorous waterfowl. We selected the top model for the focal lag based on the lowest Akaike information criterion (AIC) [57]. The range around the optimal lag was also estimated by predicting the day at which interpolated log-likelihood values had a difference of 1 (ΔAIC = 2). The difference in range was then calculated by the number of days between the minimum and maximum estimate. The predictive performance of the top models with the optimal estimated lag was evaluated by *k*-fold cross-validation [57,59]. A model with good predictive performance would be expected to be one with a high *k*-fold score (*r*_*s*_ value; see electronic supplementary material, text).

For each region and each species, two main cues could be adopted, i.e. tracking the green wave or the wave of snowmelt (related to seed availability in the Northeast China Plain). We first compared the *k*-fold score (*r*_*s*_ value) of the plant model (using the green wave as the predictor) with the mean *k*-fold score of the snow models (using snowmelt as predictors) and the one with the highest score is considered the main cue. When birds track the snowmelt, the specific predictor used in the model with the minimal absolute value of the optimal lag days was identified as the specific cue (e.g., 75% snowmelt).

## 3. Results

### (a) Herbivorous waterfowl track multiple resource waves during spring migration

**Figure 2.**
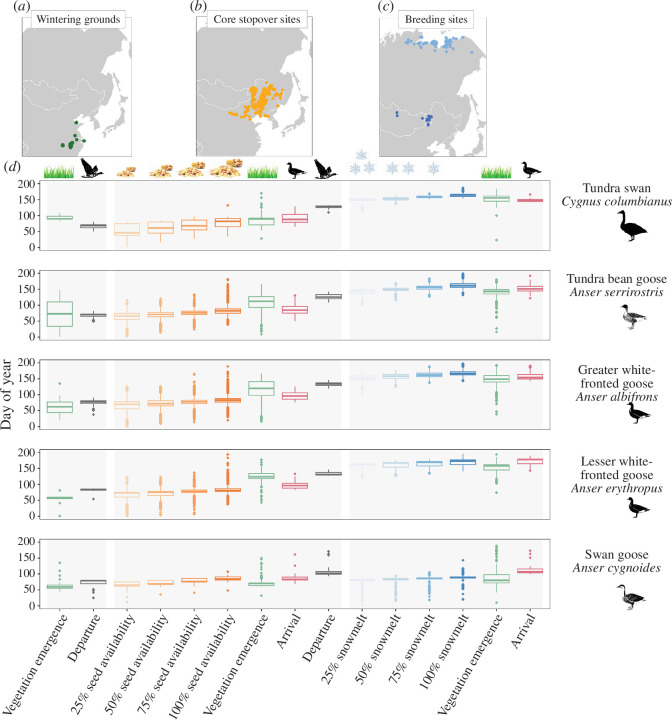
Waterfowl tracking multiple resource waves during spring migration. Dots show the centroid locations of wintering grounds (green dots in *a*), core stopover sites in the Northeast China Plain (orange dots in *b*), breeding sites in the mid-latitude (dark blue dots in *c*) and Arctic (light blue dots in *c*) used by five herbivorous waterfowl species. Greater and lesser white-fronted geese departed from the wintering grounds (black boxplots) later than other species. All study species (*d*) arrived (red boxplots) at the core stopover sites after the start of seed availability (orange, using snowmelt as a surrogate) and departed (black) from this region after the emergence of vegetation (green), thereby using both food resources. All study species arrived (red boxplots) at the breeding sites around the time vegetation emerges (green). Boxplots denote the median, upper and lower quartiles (solid boxes), and maximum and minimum values (whiskers). Transparent grey rectangles below the boxplots show different phenological events among the three regions.

Herbivorous waterfowl in East Asia adopt a strategy in which they use both the seed wave and the green wave during spring migration (figure 2 and electronic supplementary material, figure S6). All study species arrived after seeds have become available and departed after the new vegetation became available in their core stopover sites in the Northeast China Plain (figure 2, electronic supplementary material, figure S12 and table S4). The period when seeds become fully available in this region was at the end of March (mean ± s.d.: 84 ± 16 Julian days), whereas the emergence of new vegetation occured around early May (mean ± s.d.: 112 ± 30 Julian days). Birds arrived at their core stopover sites in early April (mean ± s.d.: 92 ± 15 Julian days). All Arctic-breeding species departed from the Northeast China Plain region around mid-May (mean ± s.d.: 130 ± 7 Julian days).

The specific spring migration timing varied slightly among species (figure 2, electronic supplementary material, figure S12 and figure S13). Tundra swans and tundra bean geese showed a relatively earlier departure from the wintering sites (*F* = 9.98, d.f. = 4, *p* < 0.001; figure 2, electronic supplementary material, figure S13*a* and tables S3–S4) and a relatively earlier arrival on the Northeast China Plain (*F* = 29.71, d.f. = 4, *p* < 0.001; figure 2, electronic supplementary material, figure S13*c* and tables S3–S4), in comparison to greater white-fronted geese and lesser white-fronted geese (figure 2). Swan geese departed from the wintering sites in mid-March and arrive at the core stopover sites in the Northeast China Plain around the end of March, i.e. soon after the end (100%) of snowmelt and 2 weeks after the emergence of vegetation (electronic supplementary material, table S4). The mean lag of bird arrival to seeds fully available ranges from 2 to 15 days among species, and the mean lag of departure to the emergence of vegetation ranges from 11 to 41 days (electronic supplementary material, tables S5–S9).

All study species arrived at their breeding sites around the onset of vegetation growth (figure 2, electronic supplementary material, figure S12 and table S4). Arctic breeders reached their breeding sites around early June (mean ± s.d.: 158 ± 15 Julian days; electronic supplementary material, table S3), with tundra swans arriving earlier than geese (electronic supplementary material, tables S6–S9). Lesser white-fronted geese arrived at the Arctic-breeding sites the latest compared with other species (*F =* 40.67, d.f. = 4, *p <* 0.001; figure 2, electronic supplementary material, figure S13*b* and table S3). Mid-latitude breeding swan geese reached their Inner Mongolia and Mongolia breeding sites around the end of April (mean ± s.d.: 117 ± 24 Julian days; electronic supplementary material, table S3), 3 weeks after the emergence of vegetation and full snowmelt (electronic supplementary material, table S9).

### (b) Habitat switching during spring migration

**Figure 3.**
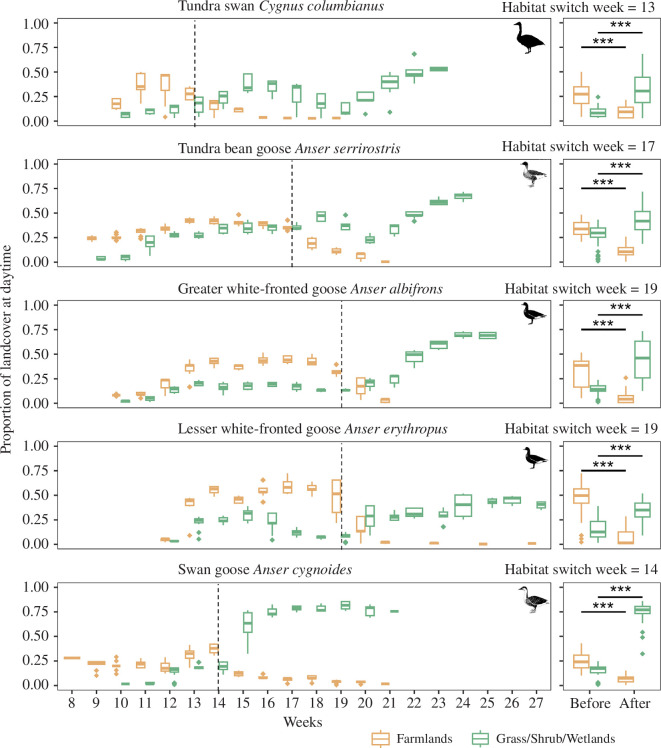
Intensity of farmland and natural habitat use during waterfowl spring migration. Boxplots indicate the proportion of habitat use in the daytime. The habitat switching week (marked with a dashed line) is defined as the week before the first week when natural habitats become utilized more intensely than farmlands. Smaller panels on the right show differences in habitat use intensity before and after the habitat switch week for five herbivorous waterfowl species. Significance is based on Welch’s two-sample *t*‐test (***p < 0.001).

Herbivorous waterfowl showed a clear habitat switch during spring migration, particularly after the emergence of vegetation at the core stopover sites in the Northeast China Plain (figure 3 and electronic supplementary material, figure S14). They spent relatively more time on farmland upon arrival and then shifted to spend more time in natural habitat after newly emerged vegetation became available (figure 3 and electronic supplementary material, figure S14). For all study species, the proportion of habitat use on farmlands in the daytime was 38% (compared with 18% on natural habitat) before the emergence of vegetation, whereas this decreased to 21% after the emergence of vegetation, resulting in a 17% shift (95% confidence interval (CI): 14–20%, Welch’s *t* = 10.14, d.f. = 268.26, *p* < 0.001) in foraging distribution from farmlands towards natural habitats (i.e. grasslands, shrublands and wetlands). This habitat shift pattern was more distinct (electronic supplementary material, figure S14) for tundra bean geese with a 16% shift (95% CI: 11–22%) and tundra swans with a 14% shift (95% CI: 6–22%). Mid-latitude breeding swan geese only arrived at the stopover area after the emergence of vegetation, and their proportion of habitat use in the daytime on farmlands (19%) was significantly lower than 53% in natural habitats (Welch’s *t* = −8.17, d.f. = 88.22, *p* < 0.001).

### (c) Cues used by waterfowl to time their arrival

**Figure 4.**
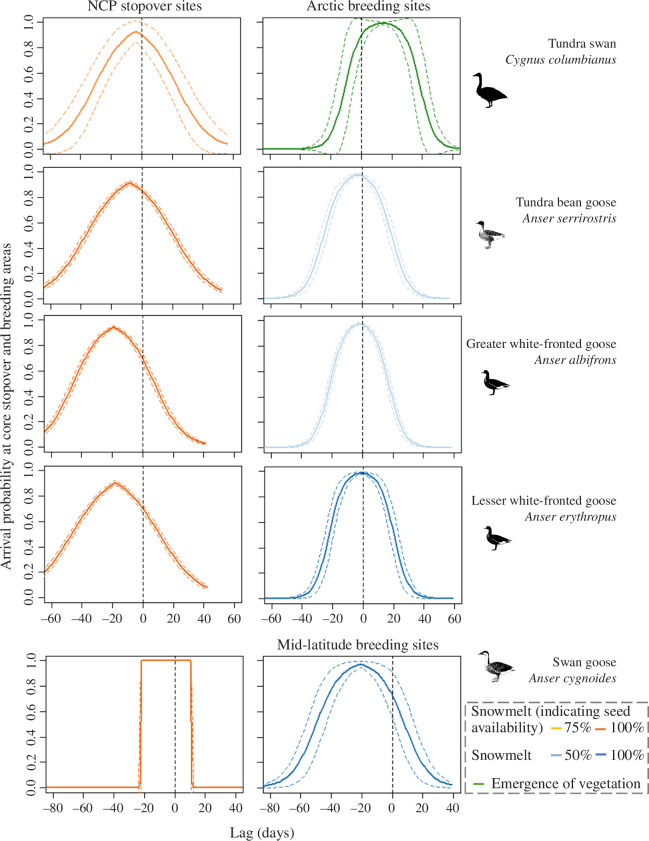
The arrival probability of East Asian herbivorous waterfowl during spring migration in relation to the availability of multiple resources. At the core stopover sites in the Northeast China Plain (NCP), all study species adopted the late (75%; light orange lines) or end phases (100%; dark orange lines) of snowmelt as the cue to track seed availability. At the breeding sites, swans tracked the onset of vegetation emergence (green lines), whereas geese tracked the mid- or end phases of snowmelt (light blue lines for 50% snowmelt and dark blue lines for 100% snowmelt). Coloured dashed lines represent 95% CIs. Vertical dashed lines represent the bird’s arrival date. Lags (*x*-axis) indicate the lag days of arrival time in comparison to the timing of resource availability or the timing of snowmelt.

The estimated minimum optimal lags among the best resource selection models (electronic supplementary material, figure S15 and table S10) evaluated by *k*-fold cross-validation (higher score and stronger effect) showed the cues that birds use in timing their spring migration (figure 4 and table 1). At the core stopover sites, the models for all study species using percentage of snowmelt (related to seed availability) as a predictor had relatively higher *k*-fold cross-validation scores. Tundra swans arrived at the core stopover sites soon after 75% snowmelt (representing 75% seed availability; 4 days; figure 4), with the range of optimal lag from 0.7 days prior to 8.4 days after this date. Geese arrived later, using the full (100%) snowmelt as a cue to track seed availability (figure 4 and table 1).

**Table 1.** Top resource selection models showing cues used by waterfowl in timing their arrival in specific regions.

region	species	scientific name	model/cue	estimated lag days with range	difference	log-likelihood
Northeast China Plain	tundra swan	*C. columbianus*	snowmelt (75% seed)	−4 (−8.4, 0.7)	9.1	−15.11
	tundra bean goose	*A. serrirostris*	snowmelt (100% seed)	−8 (−9.3, −7.6)	1.7	−52.19
	greater white-fronted goose	*A. albifrons*	snowmelt (100% seed)	−19 (−19.1, −18)	1.1	−3393.12
	lesser white-fronted goose	*A. erythropus*	snowmelt (100% seed)	−18 (−18.7, −16.8)	1.9	−1428.18
	swan goose	*A. cygnoides*	snowmelt (100% seed)	−6 (−6.2, −4.8)	1.4	−1515.25
breeding sites	tundra swan	*C. columbianus*	plant	14 (12.7, 15)	2.3	−2099.11
	tundra bean goose	*A. serrirostris*	snowmelt (50%)	−3 (−4, −2.8)	1.2	−66.86
	greater white-fronted goose	*A. albifrons*	snowmelt (50%)	−2 (−2.6, −1.8)	0.8	−1379.61
	lesser white-fronted goose	*A. erythropus*	snowmelt (100%)	−1 (−1.6, −0.6)	1	−489.40
	swan goose	*A. cygnoides*	snowmelt (100%)	−21 (−21.4, −20.4)	1	−1992.69

The estimated range represents the day at which interpolated log-likelihood values had a difference of 1 (ΔAIC = 2).

The difference is the number of days between the minimum and maximum estimate of the range.

At the breeding sites, for tundra swans, the plant model had a higher *k*-fold score than the mean *k*-fold scores of the snowmelt models (electronic supplementary material, table S10). Tundra swans thereby used the emergence of plants as a cue (figure 4 and table 1). For geese, the snow models had a higher mean *k*-fold score than that of the plant model (figure 4 and electronic supplementary material, table S10). Tundra bean geese and greater white-fronted geese used the mid of (50%) snowmelt as a cue (both *r*_*s*_ > 0.9; figure 4 *,* table 1 and electronic supplementary material, table S10), whereas lesser white-fronted geese and swan geese used snow-free time as a cue (strongest effect for 100% snowmelt predictor; figure 4, table 1 and electronic supplementary material, table S10).

## 4. Discussion

We test a new migration strategy in which East Asian herbivorous waterfowl use multiple resource waves to time their spring migration. All study species use the snowmelt as the main cue to time their arrival at the core stopover sites in the Northeast China Plain and track either the onset of spring or the mid- or end phases of snowmelt when arriving at their breeding sites. Birds mainly use farmlands after reaching the core stopover sites and only switch to mainly using natural habitats after new vegetation becomes available. These strategies, in which migration is timed to be able to take advantage of both natural and anthropogenic food resources, allow East Asian herbivorous waterfowl to exploit the vast and variable landscape along their migration route.

East Asian migratory waterfowl track the late or end phases of snowmelt at their core stopover site to profit from seed availability. Seeds left over from the previous year’s harvest on the unfrozen ground (from the beginning of April) in the core stopover sites in the Northeast China Plain provide high-nutrient food for herbivorous waterfowl, especially in early spring, when the snow has just melted and food in the natural habitat is not yet available. The newly emerged plants (in early May) allow migratory birds to replenish their energy for an extended period of time during stopover, which facilitates arrival at the breeding grounds in optimal conditions.

The three relatively larger-bodied species (tundra swans, tundra bean geese and swan geese) show an earlier and more distinct shift towards using the natural habitat than the two smaller-bodied ones (greater and lesser white-fronted geese; electronic supplementary material, figure S14). This probably reflects that the smaller-bodied species stay in the wintering grounds for an extended period of time after the emergence of vegetation, foraging on newly emerging grasses before starting their spring migration [60–62], and as a consequence, arriving much later at the core stopover and the breeding sites than large-bodied species. In contrast, larger-body herbivorous waterfowl are generally more tolerant of forage quality and therefore can utilize more diverse food resources [45,61]. Thus, they arrive at core stopover sites earlier and can better use the seeds in farmlands [63] and/or the underwater food resources including tubers and algae [41]. Smaller-bodied species arrived relatively later so they use farmland for a shorter period before moving to their natural habitat.

Waterfowl use the emergence of vegetation or the mid- or end phases of snowmelt as a cue to time their arrival at their breeding sites. The relatively larger species of tundra swans arrive at the Arctic earlier and use emerging spring vegetation as a cue. They generally invest more time in breeding activities in which they have to lay eggs, moult and raise chicks, than relatively smaller species [4,64,65]. The slower pace in their life histories (i.e. laying larger eggs with a longer incubation and gosling-rearing period) drives birds to reach the breeding grounds and lay eggs earlier [65,66]. Three Arctic-breeding goose species arrive later and use half or full snowmelt as the cue to time their arrival at the Arctic. Tracking mid- or end phases of snowmelt enables them to use the newly emerged vegetation to enable sufficient fuel to be accumulated and avoid arriving too early and depleting their body stores while awaiting the local thaw [4,6,63]. The mid-latitude breeding swan geese arrive at the breeding grounds much later than the end of snowmelt. These birds have a relatively longer breeding window compared with Arctic breeders and the snow-free condition facilitates efficient feeding on both spring grass and aquatic tubers [67].

Climate change might induce shifts in vegetation phenology in spring [68–70], thereby changing food availability for herbivorous waterfowl at a different pace at different sites [71,72]. Reduced predictability of food supply due to phenological asynchrony would be exacerbated when different food resources are exploited [72]. As a consequence, species may struggle to advance their migration timing to match food availability [73]. Even though some goose species can accelerate their migration speed under global warming, they might still suffer population declines if they cannot store sufficient nutrients for laying eggs [64].

For species taking advantage of the seed wave, migration timing and pace could be influenced by changes in cultivation practices [37]. For example, changes in farming calendars might affect seed availability [17,24,37]. Different responses to environmental change between the timing of cultivation and bird migration phenology might cause population declines of migratory birds [74,75]. Therefore, habitat management should consider conservation needs, especially for species that have come to depend on anthropogenic food resources [76]. Moreover, to what extent populations of migratory herbivorous waterfowl benefit from, or are threatened by, this migration strategy of tracking multiple resource waves needs to be thoroughly investigated. For example, how the seed wave itself is affected by climate and agricultural practices is still unknown. A proper understanding of the impact of global environmental change and different human cultivating activities on migratory bird species will guide effective habitat management for migratory bird conservation worldwide.

Satellite-derived indices provide an efficient way to measure the seed wave and green wave, given that data that directly measure food availability site-by-site in the field are costly to obtain. They also cover more detailed pixel-by-pixel information in space and time, and better match the GPS locations for tracked birds. However, satellite-derived plant emergence timing has uncertainties depending on the smoothing and retrieval algorithms used [52]. We chose the second derivative EVI method out of six retrieval algorithms to calculate the emergence of vegetation since the output date is the closest to the field observation in our study region (electronic supplementary material, figure S8). Regarding the seed wave, it is crucial to investigate how the timing of snowmelt in spring affects the actual availability of seeds and how much of those seeds are eaten by waterfowl moving through the region. Systematic ground-truth data should be used in future studies to further optimize the derived plant and seed availability estimates.

## 5. Conclusion

Our study provides the first evidence for multiple resource tracking during waterfowl spring migration. The central tenet of the resource tracking theory is that mobile consumers can benefit by moving to exploit phenological variation in resources across space [5]. Tracking multiple resource waves allows migratory herbivorous waterfowl to prolong the overall resource availability of natural and anthropogenic resources. To further our understanding of the response of species to environmental change, the capability of tracking multiple resource waves should be further tested in additional study systems and in different geographical regions. Our findings further the understanding of migration mechanisms under global environmental change and highlight conservation opportunities through managing both anthropogenic and natural habitats.

## Data Availability

All code and derived data for analyses, tables and figures have been deposited on Dryad [77]. Supplementary material is available online [78].
